# Hypocotyl Transcriptome Reveals Auxin Regulation of Growth-Promoting Genes through GA-Dependent and -Independent Pathways

**DOI:** 10.1371/journal.pone.0036210

**Published:** 2012-05-09

**Authors:** Elisabeth J. Chapman, Kathleen Greenham, Cristina Castillejo, Ryan Sartor, Agniezska Bialy, Tai-ping Sun, Mark Estelle

**Affiliations:** 1 Section of Cell and Developmental Biology, Division of Biological Sciences, University of California San Diego, La Jolla, California, United States of America; 2 Biology Department, Duke University, Durham, North Carolina, United States of America; 3 Howard Hughes Medical Institute, University of California San Diego, La Jolla, California, United States of America; Instituto de Biología Molecular y Celular de Plantas, Spain

## Abstract

Many processes critical to plant growth and development are regulated by the hormone auxin. Auxin responses are initiated through activation of a transcriptional response mediated by the TIR1/AFB family of F-box protein auxin receptors as well as the AUX/IAA and ARF families of transcriptional regulators. However, there is little information on how auxin regulates a specific cellular response. To begin to address this question, we have focused on auxin regulation of cell expansion in the *Arabidopsis* hypocotyl. We show that auxin-mediated hypocotyl elongation is dependent upon the TIR1/AFB family of auxin receptors and degradation of AUX/IAA repressors. We also use microarray studies of elongating hypocotyls to show that a number of growth-associated processes are activated by auxin including gibberellin biosynthesis, cell wall reorganization and biogenesis, and others. Our studies indicate that GA biosynthesis is required for normal response to auxin in the hypocotyl but that the overall transcriptional auxin output consists of PIF-dependent and -independent genes. We propose that auxin acts independently from and interdependently with PIF and GA pathways to regulate expression of growth-associated genes in cell expansion.

## Introduction

The plant hormone auxin has diverse roles in plant growth and development including, but not limited to, embryogenesis, cell division and expansion, root initiation, tropic responses, apical dominance, flowering, and fruit and seed development [1]. A major challenge in the field of auxin biology is to understand how a small molecule can specify such distinct changes in morphogenesis and growth throughout the life cycle of a plant. Current models suggest that auxin levels are highly regulated through changes in auxin biosynthesis, conjugation and storage, degradation, and polar transport. Auxin level is then interpreted by the auxin perception machinery resulting in tissue- and cell type-specific changes in gene expression [2], [3], [4].

Auxin regulation of transcription involves a large family (23 in Arabidopsis) of DNA-binding transcription factors called the AUXIN RESPONSE FACTORs (ARF) [5], [6]. ARFs bind to promoters of auxin-responsive genes at *cis*-elements referred to as auxin response elements (*AuxREs*) [7], [8]. A TGTCTC sequence motif first identified in the auxin-responsive *GH3* promoter from soybean was shown to recruit multiple members of the *Arabidopsis* ARF family, with TGTC being absolutely required for ARF-DNA binding [9]. However, the TGTCTC element is not found in all auxin-responsive promoters. In some cases tandem repeats of the TGTC portion of the *AuxRE* are sufficient for auxin induction [10], [11]. ARF proteins are characterized by a B3-like DNA binding domain, a middle region associated with transcriptional repression or activation, and a C-terminal domain (CTD) involved in homo- and hetero-dimerization [2], [7], [8]. The CTD region is similar to the C-terminal domains III and IV of the Aux/IAA transcriptional regulators [12].

The Aux/IAAs are a 29 member family of small nuclear proteins in *Arabidopsis* that are involved in repressing auxin-regulated transcription [13]. Aux/IAA proteins contain four conserved domains (I–IV), of which domains I, II and IV contain nuclear localization motifs. Domain III contains a sequence that is related to the βαα DNA binding domain that is required for Aux/IAA homo- and hetero-dimerization. However, there is currently no evidence that Aux/IAA proteins bind DNA directly [14], [15]. Rather, Aux/IAAs are recruited to promoters through interactions with ARF proteins that are mediated by domains III and IV of the two proteins. Domain II of Aux/IAAs is highly conserved and contains a degron motif that is important for degradation by the SCF^TIR1^ E3 ubiquitin ligase complex [12], [16]. Mutations in this degron result in stabilization of the protein and reduced auxin response, causing various defects in growth and development [6], [16], .

Functional redundancies within the ARF and Aux/IAA gene families make assigning specific roles of each protein a challenge. However, genetic studies have revealed ARF and Aux/IAA combinations that are essential for certain processes. BDL/IAA12 and MP/ARF5 specify apical-basal polarity during embryogenesis [18], SLR/IAA14 and NPH4/ARF7 are required for lateral root initiation, and MSG2/IAA19 and NPH4/ARF7 are involved in tropic hypocotyl growth [19]. ARF2, ARF8, and ARF19 are involved in root and hypocotyl growth and development, although Aux/IAA partners in these processes are not clear [20], [21], [22]. Recently, the apical-basal polarity determinant TOPLESS (TPL) was shown to act as a transcriptional co-repressor with IAA12/BDL to repress ARF5/MP transcriptional activity [23]. It has yet to be seen whether all the Aux/IAAs interact with TPL to repress the auxin response in specific developmental pathways.

Auxin exerts changes in gene expression by interacting with the TIR1/AFB family of auxin receptors. These proteins are the F-box protein subunits of SCF (Skp1/Cullin/F-box) complexes that target the Aux/IAAs for proteasome-mediated degradation [24], [25], [26]. The *Arabidopsis* genome encodes 5 proteins related to TIR1, Auxin Signaling F-Box (AFB) proteins AFB1, 2, 3, 4 and 5. Previous work has shown that, like TIR1, AFB1–5 function as auxin receptors that interact with Aux/IAA repressors in an auxin-dependent manner [26], [27]. Mutant analysis reveals overlapping functions of TIR1/AFB1–3. The most severely affected *tir1 afb1 afb2 afb3* quadruple mutants arrest shortly after germination [26]. The AFB4 clade of receptors, including AFB4 and AFB5, display a unique affinity for the synthetic auxin picloram. The *afb5-5* single mutant shows almost complete resistance to picloram-induced hypocotyl growth [27].

In order to develop successful models for auxin regulation of growth and development, it will be important to identify the gene targets of the TIR1/AFB pathway(s) and understand their function in cell growth. Several studies of auxin-responsive transcriptomes have identified large numbers of candidate auxin targets. The results of supporting genetic studies ascribe developmental roles to a small number of these [28]. A potential barrier to identification of distinct auxin pathways from such studies lies in the complexity of the tissue sampled for the experiment. Auxin mediates distinct responses in different tissue types, for example inhibiting primary root elongation while stimulating lateral root initiation and outgrowth [29]. Therefore, auxin-responsive transcriptomes in entire plants are too complex to facilitate separation of distinct developmental pathways.

In this study we focus on the role of auxin signaling in cell expansion. We chose the hypocotyl, which grows entirely by cell expansion, as a model tissue for this study [30]. The hypocotyl elongates in plants overexpressing auxin biosynthetic genes [31] and in response to high temperature [32], due to elevated auxin levels. Hypocotyl elongation is tightly regulated and many signaling pathways overlap to regulate uniform, as well as directional, hypocotyl cell expansion. Light is a major repressor of hypocotyl growth and as a consequence, mutations in the phytochrome light receptors result in seedlings with long hypocotyl phenotypes [33]. Light-activated forms of the phytochromes interact with members of the phytochrome-interacting factor (PIF) family of bHLH transcription factors, signaling rapid phyA- and phyB-mediated degradation of PIF3,4 and 5 in the light [34], [35], [36]. PIFs have also recently been shown to function in GA signaling [37]. The PIFs appear to be the major positive regulators of hypocotyl growth, as they are required for growth responses to time of day, direction of light source, nutrients, high temperature and other stimuli [38], [39], [40], [41]. PIF mRNA and protein levels are controlled by the circadian clock, light, and GA signaling, such that PIF activities and hypocotyl growth are repressed during the day [39], [42], [37]. Within the PIF family, several *PIF* and *PIF-LIKE (PIL)* genes are implicated in germination and early seedling growth [43]. PIF4 and PIF5 seem to be particularly important for hypocotyl growth as expression of these factors is circadian regulated and correlates with hypocotyl growth [39], [42]. In addition the *pif4pif5* double mutant has a short hypocotyl phenotype [36].

Here we identify auxin signaling components required for auxin-responsive hypocotyl elongation. In addition we characterize the auxin transcriptome specifically in elongating hypocotyl tissue. Our findings indicate that auxin-induced hypocotyl elongation is associated with regulation of a suite of growth-associated genes and involves GA biosynthesis. Importantly, we also show that auxin works in part through pathways independent of GA and PIF activities.

## Results and Discussion

### Auxin Promotes Elongation of *Arabidopsis* Hypocotyls

**Figure 1:**
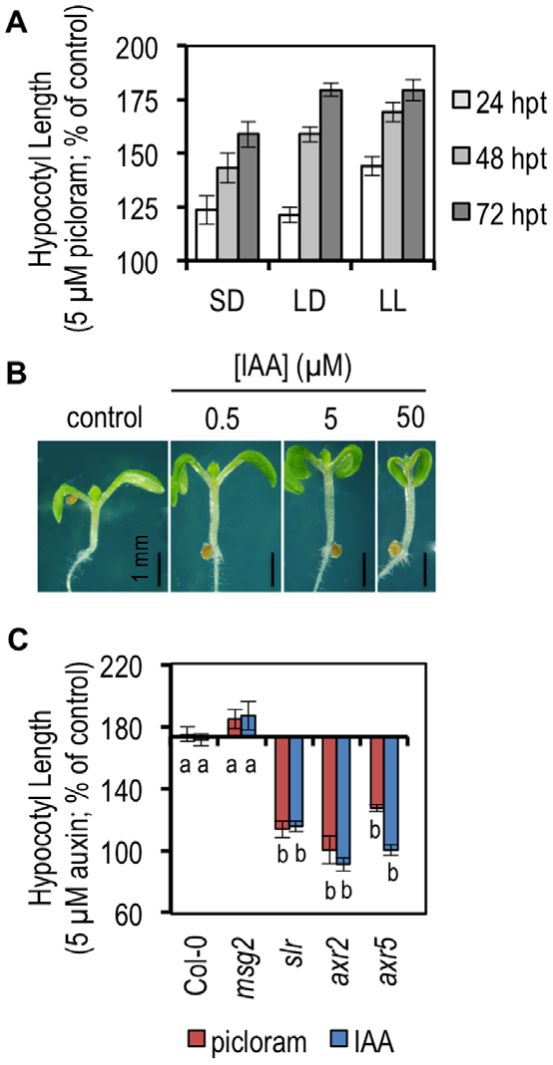
Auxin promotes hypocotyl elongation in light-grown seedlings. Auxin promotes hypocotyl elongation in a range of day-length conditions. Average hypocotyl length of wild-type seedlings grown in short days (SD; 8/16), long days (LD; 16/8) or constant light (LL) and treated with 5 µM picloram was determined following 24, 48, or 72 hours of auxin treatment. Hypocotyl length on auxin is shown as a percentage of length on control medium. Error bars indicate standard error. (B) Auxin response in seedlings increases with auxin concentration. Images of aerial portions of individual 7 day-old seedlings were captured following 48 hours of IAA treatment at the indicated concentrations. (C) Hypocotyl auxin response requires auxin signaling. Average hypocotyl length of wild-type or *aux/iaa* mutant seedlings treated with 5 µM picloram (red bars) or IAA (blue bars) was measured following 48 hours of auxin treatment. Hypocotyl length on auxin relative to the untreated control is shown as in (a). *Error* bars indicate standard error. Statistical significance was determined using a Tukey HSD post hoc comparison among the means on the analysis of variance using type III sums of squares (p<0.05).

**Figure 2:**
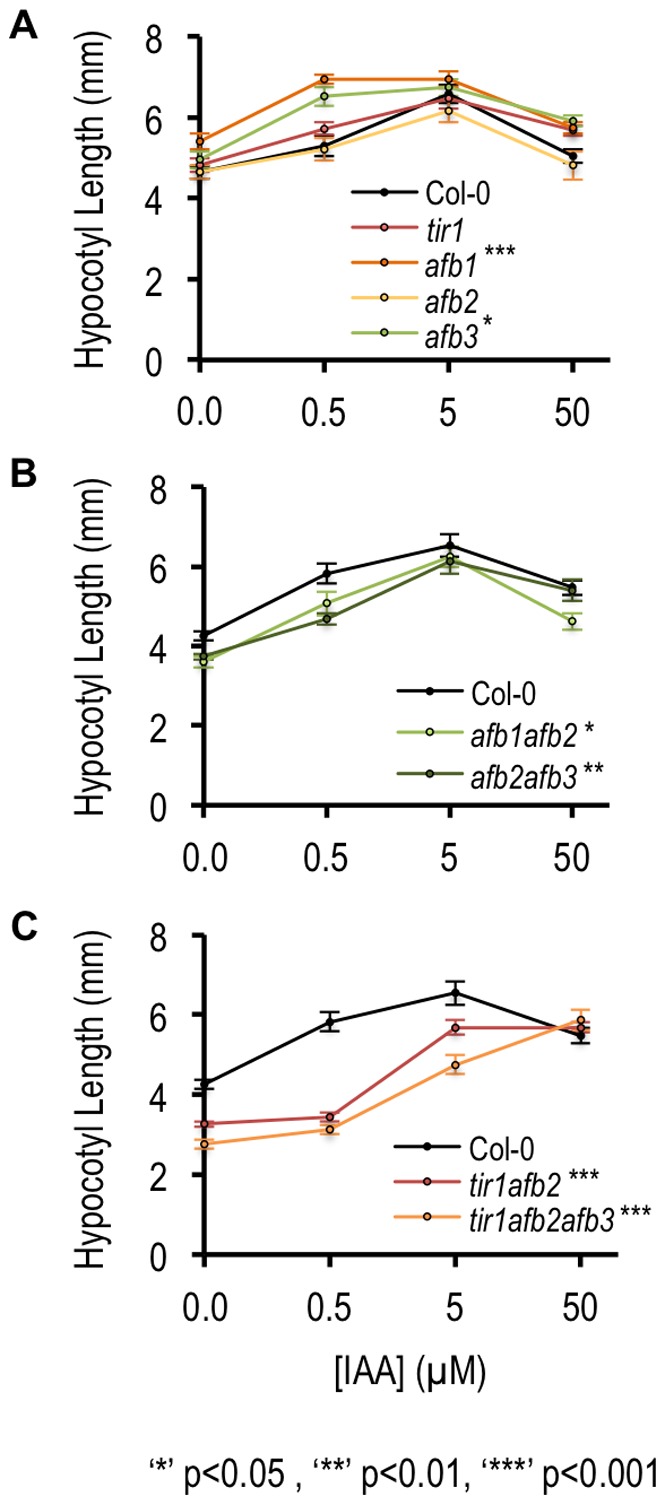
Hypocotyl auxin response requires TIR1/AFB auxin receptors. (A–C) Hypocotyl length of wild-type or *tir1/afb* single or multiple mutant seedlings grown in short days and treated with IAA at the indicated concentrations was measured following 48 hours of auxin treatment. Asterisks represent mutants showing a significantly different response to hormone treatment compared to wildtype. A general linear model (glm performed in R using the car package [104]) was used to determine significance and main effects for genotype were confirmed using ANOVA type III sums of squares. All assumptions for GLM were fulfilled. Error bars indicate standard error.

To explore the function of auxin in plant growth, we have elected to focus on the *Arabidopsis* hypocotyl. Our first task was to develop a robust assay for auxin response in this system. Seedlings were grown at 22C for 5 days in various day-night cycles, exposed to auxin, and measured after different treatment times. Initially, we treated seedlings with the synthetic auxin picloram because earlier studies showed that this compound promoted hypocotyl elongation while the natural auxin indole acetic acid (IAA), generally inhibited elongation [27], [44], [45]. However, under our conditions we found that both picloram and IAA promoted hypocotyl growth in continuous light (LL), long days (LD), or short days (SD) (Fig. 1A). Unless otherwise stated, LD conditions were used for additional experiments designed to characterize auxin-responsive hypocotyl growth (Fig. 1B). Importantly, in our conditions the auxin dose-response curve for hypocotyl growth of wild-type seedlings is bell-shaped (Fig. 2). This differentiates our growth conditions from those in which auxin treatment or constitutive auxin signaling inhibits hypocotyl elongation [22], [44], [46]. Interestingly, the bell-shaped response curve is similar to auxin dose response in root system growth modeled previously [47], suggesting that an auxin signaling level optimal for eliciting a growth response may be a common feature among auxin-responsive tissues.

### Auxin-mediated Hypocotyl Elongation Requires Transcriptional Auxin Signaling

To confirm that the auxin-dependent elongation response requires activation of transcriptional auxin signaling pathways, we measured the response in a series of *Aux/IAA* gain-of-function mutants in which auxin-regulated transcription is repressed [48], [49], [50], [51], [52]. In *slr-1/iaa14, axr2-1/iaa7* and *axr5-1/iaa1*, the auxin response was significantly reduced compared to wild-type plants (Fig. 1C). Interestingly, the response of *msg2-1/iaa19* seedlings was similar to that of wild type, even though this mutant is deficient in tropic growth in the hypocotyl. This suggests that different auxin signaling pathways have specific roles in hypocotyl growth. This has been shown previously for apical-basal polarity determination [18] and lateral root initiation [21].

We explored the possibility of functional specialization among the TIR1/AFB auxin receptors in hypocotyl elongation by analyzing the phenotypes of various tir1/afb mutants. We observed slight auxin resistance or hypersensitivity in single tir1/afb receptor mutants (Fig. 2A) with the exception of *afb5-5* and *afb4-2 afb5-5,* which are highly resistant to picloram [27]. The basis for auxin hypersensitivity in afb1-3 and afb3-4 mutants is unclear, however, double mutant combinations among afb1-3, afb2-3, and afb3-4 eliminated this hypersensitivity (Fig. 2B) suggesting that increased growth response may be due to enhanced activity of other TIR1/AFB family members that is lost in the higher order mutants. The *afb3-5* mutant overexpresses *AFB1* and *AFB2* due to alterations in small RNA-mediated regulation, and *afb2-3* overexpresses *AFB1* and *AFB3* [53]. Thus, TIR1/AFB single mutants may not display predictable loss-of-function phenotypes. Future analysis of the expression patterns of the receptors in the single and double mutant backgrounds will be necessary to determine whether elevated receptor activity in TIR1/AFB mutants could explain the hypersensitivity we observed.

Double and triple mutants carrying *tir1-1* each displayed increased auxin resistance when compared to the *tir1-1* mutant (Fig. 2C). The triple mutant *tir1-1 afb2-3 afb3-4* displays an incompletely penetrant phenotype in which a significant percentage of individuals fail to develop basal structures such as roots and hypocotyls [26]. In *tir1-1 afb2-3 afb3-4* individuals with developed basal structures, hypocotyls were shorter than those of wild-type plants and displayed the highest degree of resistance to IAA-mediated elongation of all *tir1/afb* receptor mutants (Fig. 2C). The reliance of the elongation response on the TIR1/AFB auxin receptors and degradation of Aux/IAA proteins confirms that auxin mediated growth requires transcriptional auxin signaling pathways.

### Identification of Auxin-responsive Cell Expansion-associated Genes in Elongating Hypocotyls

Based on our finding that auxin-mediated hypocotyl elongation requires the TIR1/AFB pathway, we hypothesized that elongation is preceded by changes in expression of a suite of auxin-responsive genes. To identify such genes, we profiled auxin-responsive transcription in hypocotyls in a series of microarray experiments. We incorporated several parameters into our microarray design to maximize the likelihood of identifying auxin-regulated genes associated with anisotropic cell expansion. To enrich our dataset for cell expansion genes that may not be identified in whole seedling experiments, we sampled hypocotyl tissue dissected from auxin- or control-treated whole seedlings. To minimize time-of-day and circadian effects and avoid mis-identification of auxin-responsive genes, we treated experimental and control seedlings at the same time of day and limited the dissection time to 30 minutes. To maximize the amplitude of the transcriptional auxin response, we treated seedlings two hours after subjective dawn, when hypocotyl growth is minimal in the absence of exogenous auxin [54]. Finally, we used the synthetic auxin picloram and included the *afb5-5* mutant in our microarray design, as this mutant is picloram-resistant but does not otherwise display obvious growth defects [27], [55]. We theorized that cell expansion-associated genes differentially expressed in wild-type hypocotyls elongating in response to picloram might not be responsive in *afb5-5* hypocotyls, which fail to elongate in response to picloram.

**Figure 3:**
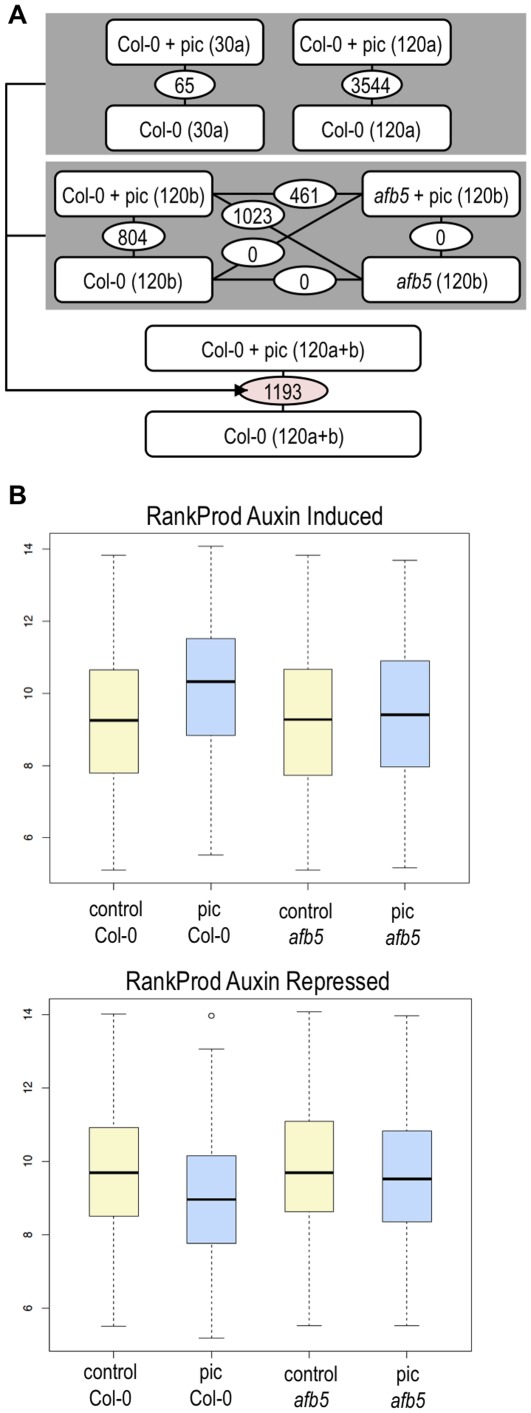
The *afb5* mutant fails to respond to picloram. The *afb5-5* mutant fails to regulate transcription in response to picloram. Differential gene expression between hypocotyl samples of solvent-treated wild-type (Col-0) or *afb5-5* seedlings (*afb5*) or seedlings treated with picloram (+ pic) for 30 minutes (30) or 120 minutes (120), as determined by analysis of microarray data. The number of genes differentially expressed between samples is shown in lines connecting each sample pair. Data from microarray experiments (a) and (b) were combined for identification of 1193 picloram-responsive genes. (B) Aver*age e*xpression values of 740 auxin-induced (upper panel) or 453 auxin-repressed (lower panel) genes are not different in hypocotyls from control seedlings (Col-0), co*ntrol-t*reated *afb5-5* mutant seedlings (control *afb5*) or picloram-treated *afb5-5* mutant seedlings (pic *afb5*). Differentially expressed genes identified using the RankProd package were selected and average expression values for microarray ‘b’ are shown.

For microarray experiment “a”, we sampled hypocotyls from wild-type plants treated for 30 minutes or 2 hours with picloram or a solvent-only control. For experiment “b”, we sampled hypocotyls from wild-type or *afb5-5* plants treated for 2 hours with picloram or a solvent-only control (Fig. 3A, Table S1). Following auxin or control treatment of seedlings, hypocotyls were individually dissected and frozen for subsequent RNA isolation.

To identify genes differentially expressed among the treatments, we used a moderated linear model [56] and an FDR cutoff of <0.05 to filter data from each microarray experiment. From this initial analysis we identified 65 genes differentially expressed following the 30-minute auxin treatment (Table S2), and 3544 (experiment “a”) or 804 (experiment “b”) genes differentially expressed following a 2-hour auxin treatment (Fig. 3A). Consistent with the picloram-resistant phenotype of *afb5-5*, no differential expression was detected in *afb5-5* following picloram treatment using the analysis method described. Interestingly, we were also unable to identify genes differentially expressed between wild-type and *afb5-5* untreated samples (Fig. 3A). So far, picloram perception and regulation of picloram-responsive transcription is the only known function of the AFB5 auxin receptor. The identification of additional functions for AFB5 will require alternative experimental approaches. Analysis of genes differentially expressed in wild-type hypocotyls following 30 minutes of picloram treatment indicated that *SAUR* genes, *AUX/IAA* genes, *GH3* genes and others shown elsewhere to be early auxin-responsive [57] were induced by picloram and were the predominant genes to be regulated at this time-point (Table S2). For additional insight into gene expression associated with auxin response, we focused on data from the 2-hour time-point samples.

Comparison of gene lists from the 2-hour auxin treatment in experiments “a” and “b” identified 267 genes differentially expressed in both experiments. This modest overlap may be due to experimental variables such as differences in RNA extraction methods and microarray hybridization parameters, or perhaps more importantly, to ‘lab-effects’ such as those previously shown to serve as a source of variability among microarray experiments performed on the same platform at different laboratories [58] (see Methods). To increase the validity and statistical strength of the comparison we used the RankProd package in R that accepts pre-processed data generated from different laboratories and platforms [59]. This package is an extension of the rank product method that implements a non-parametric statistic to compare the expression-based rankings of genes across samples [60]. From this analysis, we identified 1193 genes differentially expressed between control and 2 hour auxin-treated samples; 740 of these genes are induced, and 453 are repressed by picloram (Table S3). The mean expression levels of these two gene sets in microarray “b” are not affected by picloram treatment of *afb5-5* mutant plants (Fig. 3B), suggesting that these are indeed downstream targets of picloram-stimulated transcriptional auxin signaling. We focused on the set of 1193 candidate auxin-responsive cell expansion-associated genes for all additional experiments.

### Picloram and IAA Regulate a Common Set of Target Genes

The synthetic auxin picloram induces a hypocotyl elongation response similar to that observed with IAA, suggesting that the downstream targets of picloram- and IAA-stimulated auxin signaling are common between these two auxin pathways. The failure of *afb5-5* to regulate this set of genes or to elongate in response to picloram is consistent with a model in which the genes are targets of auxin signaling and involved in the elongation response. To confirm this, we performed a comprehensive comparison between our auxin-responsive gene set and publicly available microarray data. Our first comparison was done using the MASTA package available from the BAR website (http://bar.utoronto.ca/welcome.htm) that probes differentially expressed genes against a database of 600 contrasts obtained from publicly available microarray datasets. Of the 740 genes upregulated by picloram in our dataset, 219 were identified as auxin-upregulated in IAA treatment arrays; of 453 genes downregulated by picloram in our dataset, 121 genes were identified as auxin-downregulated in IAA arrays (data not shown). These overlaps are statistically significant (p.value <0.001) and confirm that picloram affects known IAA-responsive genes. We also performed independent comparisons with the Nemhauser *et al.* [61] and Stepanova et al. [62] auxin treatment datasets (see Methods for details of comparison). In both cases, more than 50% of the IAA-induced genes were induced by picloram in our experiments (Fig. S1A). The Stepanova *et al.* [62] dataset was obtained from experiments using root tissue suggesting that many of the genes involved in hypocotyl growth are common to root tissue. We would expect these genes to be specifically involved in cell elongation during root growth.

Importantly, our analysis identified many genes that are not presented in other auxin transcriptome datasets (Table S4) [61], [62]. Of the 740 induced genes, 521 were not described in the Nemhauser *et al.* (62) and Stepanova *et al.* (63) datasets or the 7 IAA treatment arrays found in the MASTA database. Eighty-one of these are not represented on ATH1 chips and not well characterized as auxin responsive. We expect that many of the remaining 440 genes are specifically auxin regulated in the elongating hypocotyl and were not identified in other studies because of the relative complexity of the auxin response in seedlings and roots. Similarly, 332 of the 453 repressed genes had not been described in these other datasets, 68 of which are on the Nimblegen chip but not ATH1.

To further validate the effects of picloram on auxin-responsive genes, we confirmed that a set of auxin “marker” genes, proposed to serve as hallmarks of auxin activity [61], were identified as picloram-responsive in our microarray data analysis. Overall, expression of the marker genes was responsive to picloram in wild-type hypocotyls, but not in hypocotyls from *afb5-5* mutant plants (Fig. S1B). We further validated the picloram response of several of these genes, *GH3.3, GH3.5, HAT2, IAA5, IAA19 and SAUR15*, by quantitative RT-PCR using wild-type and *afb5-5* hypocotyls. Expression of each gene was induced in wild-type hypocotyls by picloram treatment, and induction was dependent upon AFB5 (Fig. S1C). This indicates that picloram and IAA regulate an overlapping set of target genes, although the picloram signal is uniquely transduced by AFB5.

Finally, we analyzed our picloram-responsive gene set for association with auxin Gene Ontology terms and overrepresentation of *AuxRE*-containing promoter elements. GO terms associated with auxin response and hormone signaling are enriched in the annotations of our auxin-responsive gene set (Table S5), and we identified several overrepresented *AuxRE*-containing promoter elements in the promoter gene set (Fig. S2). From these results we conclude that picloram regulates the same downstream transcriptional targets as IAA, and therefore promotes hypocotyl elongation through the same transcriptional pathways as IAA. For the remaining experiments, we used picloram and IAA interchangeably or in parallel, and we did not observe qualitative differences in responses to these two auxins.

### A Profile of the Transcriptional Auxin Response Preceding Hypocotyl Elongation

**Figure 4:**
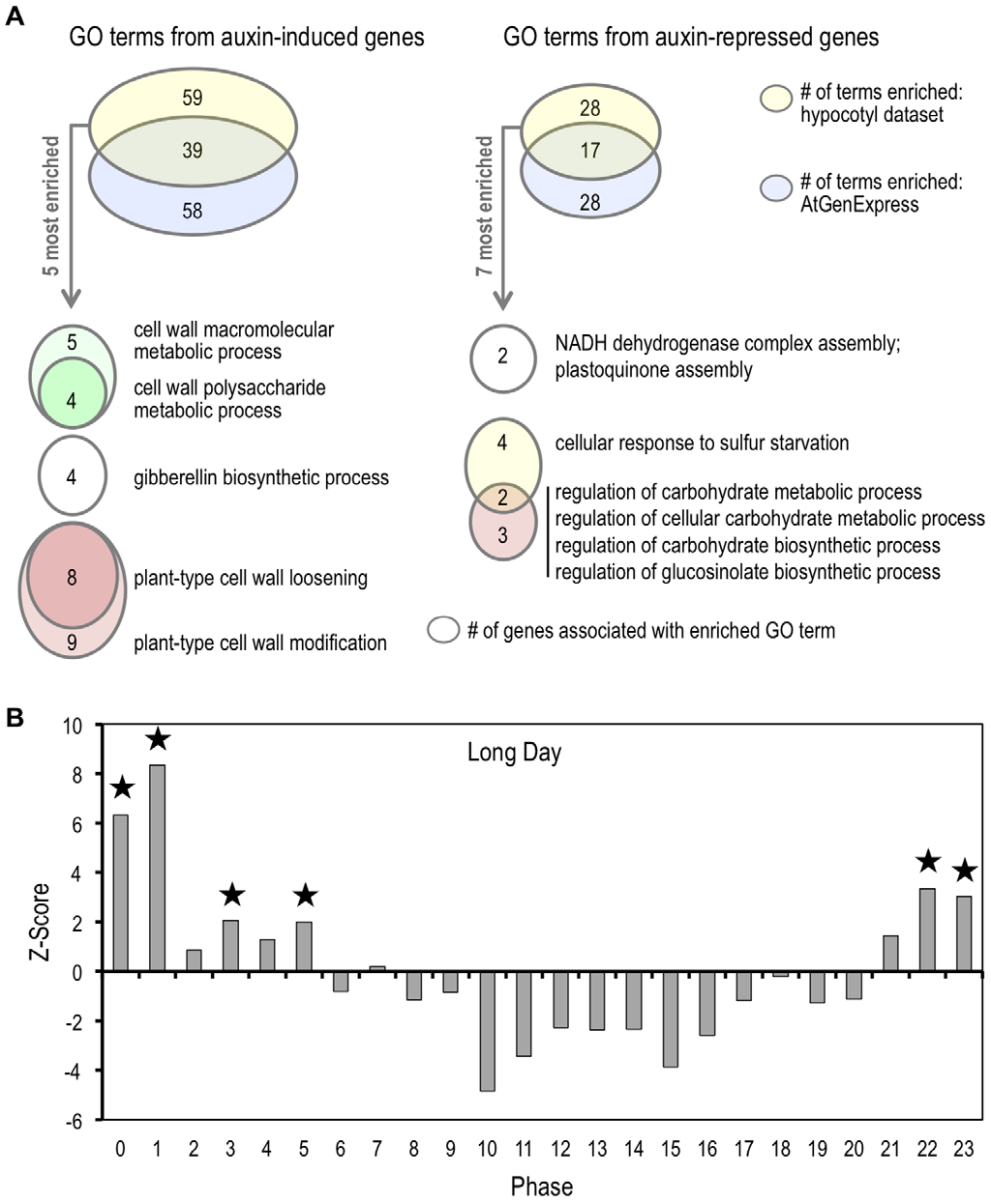
Auxin regulates a suite of growth-associated genes preceding hypocotyl elongation. Gene Ontology (GO) term enrichment in the hypocotyl datasets is similar to an IAA-responsive dataset but includes novel categories. Venn diagrams indicating the number of enriched GO terms in the auxin-induced or *-repres*sed hypocotyl datasets or *IAA* datasets from the AtGe*nExpres*s project [61] are shown. The top-ranked GO terms unique to the hypocotyl dataset are shown in the lower set of Venn diagrams. (B) Picloram induced genes are circadian regulated. The top 400 statistically significant picloram induced genes were analyzed using the Phaser tool (http://phaser.cgrb.oregonstate.edu/). Bars represent z-scores for the enrichment of cycling genes within our gene list compared to all the genes shown to cycle under long day conditions at a given phase of the day. Phase 0 signifies the start of the day. Asterisks indicate significant enrichment with a p<0.05.

Further examination of GO terms associated with our auxin-responsive gene set revealed overrepresentation of genes involved in cell wall maintenance, cell expansion, growth and hormone signaling (Fig. 4A, Table S5, Fig. S3). Enriched GO terms associated with the auxin-induced gene set included cell wall metabolism and gibberellin biosynthesis. Terms associated with the auxin-repressed gene set included carbohydrate metabolism and plastoquinone assembly (Fig. 4A). Representation of these GO processes in our auxin-responsive gene set is consistent with a role for auxin in transcriptional regulation of cell expansion-associated genes. Cell expansion in the hypocotyl, as well as in other growing plant tissues, is gated by the circadian clock and shows non-uniform patterns across a 24-hour period [63], [54], [39]. This is likely due in part to circadian patterns of expression of many genes involved in auxin signaling, biosynthesis and transport, and varying sensitivity to auxin at different times of day [64]. We theorized that genes we found to be auxin-responsive in elongating hypocotyls may follow circadian expression patterns. To determine whether circadian-regulated genes are overrepresented in our auxin-responsive gene set, we generated a gene subset consisting of the top 400 auxin-induced genes according to statistical significance, and analyzed this subset using the Phaser tool (http://phaser.cgrb.oregonstate.edu/) [63]. We observed significant enrichment of genes showing peak expression during phases 0–2 and 22–23 in LD conditions, during which hypocotyl growth is active (Fig. 4B) [63]. We further explored our auxin-induced gene set for additional determinants of expression profile by analyzing the corresponding promoter set for overrepresented regulatory elements. Interestingly, the predicted MYC/MYB binding site ‘CACATG’ was the most highly overrepresented element identified in this analysis (data not shown). The ‘CACATG’ element was previously identified as the Hormone Up at Dawn (HUD) element enriched in promoters of genes responsive to phytohormones and showing peak expression levels during periods of growth [63]. Together, these findings suggest that auxin promotes hypocotyl growth by regulating expression of cell expansion-associated genes whose expression levels are controlled by the circadian clock. This is consistent with auxin gating by the clock to maintain the diurnal pattern of hypocotyl elongation under normal growth conditions [64].

### Auxin-mediated Hypocotyl Elongation Requires GA Signaling

**Figure 5:**
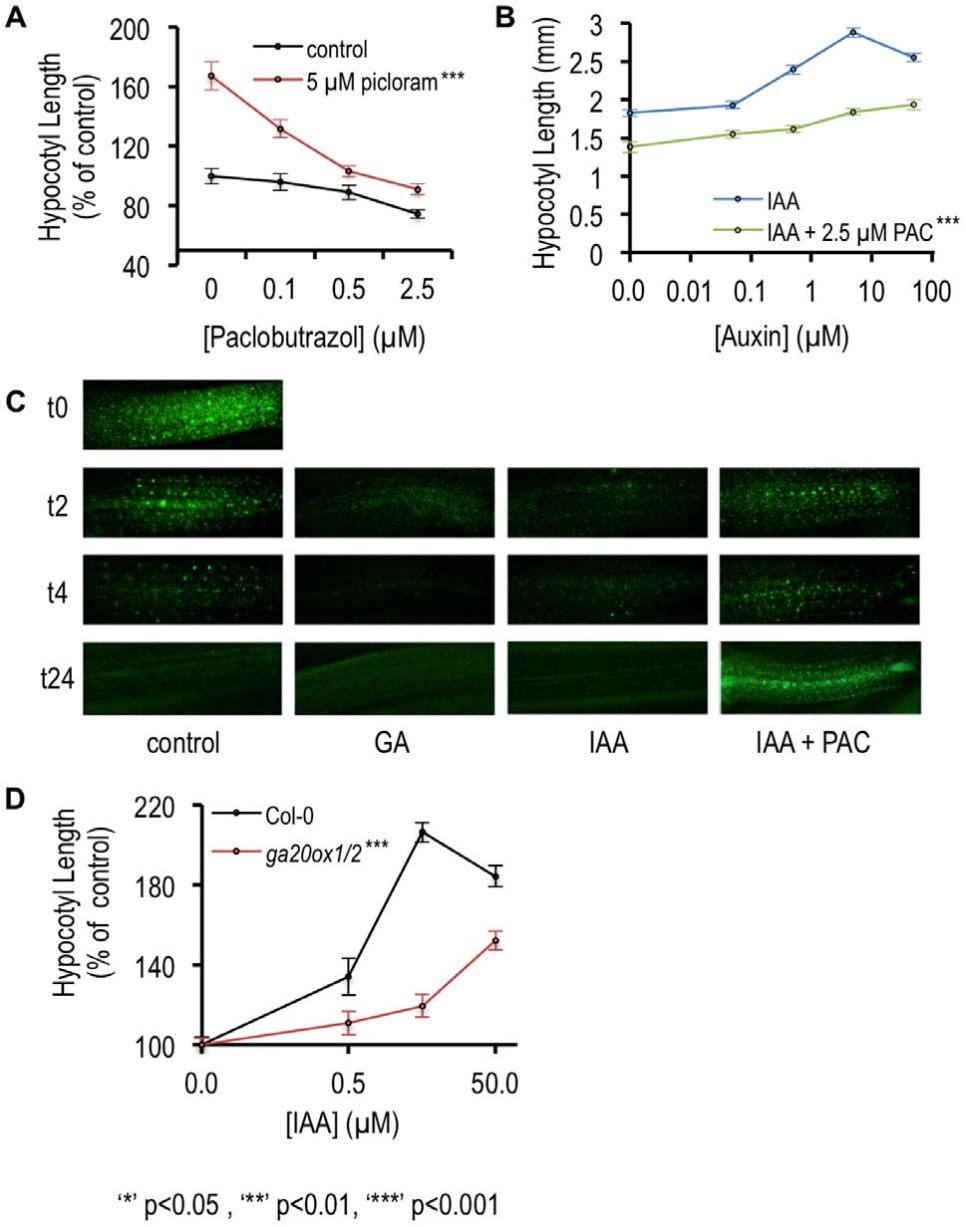
Gibberellin biosynthesis is required for hypocotyl auxin response. (A,B,C) Asterisk represents mutants showing a significantly different response to hormone treatment compared to wildtype or control treatment. A general linear model (glm performed in R using the car package [101]) was used to determine significance and main effects for genotype were confirmed using ANOVA type III sums of squares. All assumptions for GLM were fulfilled. (A)_Paclobutrazol inhibits hypocotyl auxin response. Hypocotyl length of wild-type seedlings grown in short-day conditions and treated with paclobutrazol at the indicated concentrations (black line) or paclobutrazol plus 5 µM picloram (red line) was measured following 48 hours of treatment. Error bars indicate standard error. (B) Paclobutrazol-mediated inhibition of hypocotyl elongation is not overcome by higher auxin concentration. Hypocotyl length of wild-type seedlings treated with IAA (blue line) or picloram (red line) at the indicated concentrations or IAA and 2.5 µM paclobutrazol (PAC; green line) was measured following 48 hours of treatment. Error bars indicate standard error. (C) RGA protein degrades in response to auxin treatment in hypocotyls of auxin-treated seedlings. Abundance of RGA-GFP protein in hypocotyl tissues was analyzed by epifluorescence microscopy over a 0–24 hour time course. Three day-old seedlings were treated for 2, 4, or 24 hours with 50 µM GA_3_, 5 µM IAA, 5 µM IAA +2.5 µM paclobutrazol, or a solvent control, prior to imaging. (D) A GA biosynthesis mutant is partially auxin-resistant. Hypocotyl length of wild-type seedlings (Col-0) or the *ga20ox1/ox2* mutant [98] treated with IAA at the indicated concentrations was measured following 48 hours of auxin treatment. Hypocotyl length on auxin is shown as a percentage of length on control medium. Error bars indicate standard error.

A number of studies have shown that auxin and GA interact to regulate elongation growth in stems and hypocotyls [65], [66], [67], [68], [69]. For example, in pea stem and Arabidopsis seedlings, auxin regulates the expression of a number of GA metabolic genes including members of the *GA20OX* and *GA3OX* gene families, involved in synthesis of active GAs, as well as *GA2OX* genes, involved in GA inactivation [66], [70]. In addition, Frigerio et al [66] showed that the long hypocotyl phenotype conferred by overexpression of *YUCCA1* is suppressed by the GA biosynthesis inhibitor paclobutrazol, indicating that GA synthesis is required for auxin-dependent hypocotyl growth. We found that *GA20OX1*, *GA20OX2*, *GA2OX8*, and *GA3OX1* are auxin-regulated in the hypocotyl (Table S2, Table S3). To expand on the role of GA biosynthesis in auxin-mediated hypocotyl elongation, we tested the effect of adding paclobutrazol to auxin treatment assays. Paclobutrazol inhibited the effects of exogenous auxin in our system, as co-treatment with paclobutrazol attenuated, but did not abolish, the hypocotyl elongation promoted by picloram (Fig. 5A) or IAA (Fig. 5B). This suggests that active GA biosynthesis is required for optimal hypocotyl auxin response, consistent with earlier reports [66].

These results are consistent with a model in which increased auxin levels promote an increase in GA levels, and this GA increase is required for the elongation response. Auxin and GA are known to be involved in the effects of elevated temperature on hypocotyl elongation. Temperature-mediated elongation depends upon auxin biosynthesis [32], [71], [72]. Stavang et al. [69] showed that the temperature response also requires GA and that both *GA20ox1* and *GA3ox1* are up-regulated at higher temperature. They concluded that GA and auxin act independently, based on the behavior of pentuple *della* mutants [69]. However, it has also been shown that co-treatment of seedlings with GA and NPA attenuates the GA response [44]. These results suggest that auxin and GA act interdependently to regulate hypocotyl length.

We further examined our microarray data for overlap between the auxin/GA pathway we present here and the temperature-response pathway mediating hypocotyl elongation. Interestingly, we find that our hypocotyl auxin-regulated gene set is quite distinct from the gene set responding to elevated temperature in seedlings. Of the 113 temperature up-regulated genes presented by Stavang *et al.*, only 13 are also induced by auxin in the hypocotyl [69]. These findings suggest that most transcriptional changes associated with temperature are not related to auxin, or may occur predominantly in non-hypocotyl seedling tissues.

GA acts by stimulating the degradation of growth repressing proteins called the DELLAs [73]. Previous work has shown that auxin promotes degradation of the DELLA proteins in the root and that this degradation is required for GA regulated root growth [74]. However, how auxin regulates DELLA levels is not clear. One possibility is that loss of the DELLAs is caused by an auxin-dependent increase in GA levels. To determine if this might be happening in the hypocotyl, we tested the effects of exogenous auxin on stability of the DELLA protein RGA. Treatment of seedlings expressing RGA-GFP with IAA or GA resulted in loss of RGA protein from hypocotyl cells within 2 hours (Fig. 5C). This auxin effect was abolished by co-treatment with paclobutrazol (Fig. 5C). While it is possible that the observed loss of RGA protein in auxin-treated seedlings is due to an effect of auxin on transcription of *RGA*, we think this is unlikely as we did not identify *RGA* as an auxin-downregulated gene in our microarray experiments (although we did identify *RGA-LIKE1* (AT1G66350) and *RGA-LIKE3* (AT5G17490) as auxin-upregulated genes, see Table S3). DELLA protein abundance is also affected by circadian regulation of GA signaling [75]. However, it is unlikely that circadian regulation fully explains the effects we observed on RGA-GFP levels, as DELLA protein levels increase during the day [75] where we observed a decrease. A more likely possibility is that auxin regulation of GA levels results in degradation of RGA-GFP protein in the seedlings. We did observe that the RGA-GFP signal decreased in the hypocotyl throughout the course of the experiment, and therefore that the signal in control seedlings was weaker at the 24-hour time point than at time zero. This is also unlikely to be due to circadian patterns, which follow a 24-hour cycle. It is possible that the overall abundance of RGA-GFP in hypocotyls changes with growth dynamics, and that more sensitive imaging methods could be used to visualize the protein in older tissues.

We further explored the requirement for GA biosynthesis and signaling in auxin response by examining the behavior of the *ga20ox1 ga20ox2* double mutant in the hypocotyl elongation assay. Plants compromised in endogenous GA levels due to mutations in *GA20OX1* and *GA20OX2* showed partial auxin resistance (Fig. 5D). These data suggest that auxin and GA act interdependently in hypocotyl cell expansion.

We noted, however, that in our hormone treatment assays, paclobutrazol did not completely abolish the auxin effect (Fig. 5A, 5B). While this inhibitor may not fully suppress GA accumulation in the seedlings, our results suggest that the elongation-promoting effects of auxin may not be limited to regulation of GA metabolism. We further explored the auxin-GA interaction by testing the ability of GA to restore the short hypocotyl phenotypes of several gain-of-function Aux/IAA mutants. We found that GA did not significantly affect the hypocotyl length of the *axr2* mutant, and that the hypocotyl phenotypes of these mutants overall were only partially restored by treatment with GA (Fig. S4A). These data indicate that auxin signaling is required for a growth program independent of the regulation of GA metabolism, and that constitutive repression of auxin signaling in the Aux/IAA mutants represses this program. We propose that auxin promotes hypocotyl growth in part through GA and in part through an unknown independent pathway(s). A mechanism by which auxin can induce hypocotyl growth independently of GA synthesis may be important for rapid growth responses.

It is important to note that while the results of our paclobutrazol experiments are consistent with a model in which auxin stimulates synthesis of GA, which then contributes to the elongation response, this may be an oversimplification. GA levels are under negative feedback regulation in which expression of GA biosynthesis genes is repressed as GA levels increase [76]. Auxin biosynthesis in turn is regulated in part by PIF4, which is indirectly activated by GA. Dynamic regulation between these two hormone pathways is likely to be important for hormone-mediated cell expansion.

### Auxin Promotes Cell Expansion Independent of Time of Day in Part through Regulation of PIF-independent Pathways

As previously mentioned, several signaling pathways are important for controlling hypocotyl growth, including light signaling and the circadian clock, as well as hormone signaling [37], [39], [42]. Many of the growth-associated downstream genes in these pathways are regulated by PIF transcription factors [37], [39], [42], recently shown to be required for activation of transcription downstream of GA signaling [37], [39], [42]. PIF4 and PIF5 are two members of the PIF family that are circadian regulated and for which expression level is correlated with hypocotyl growth [39], [42], [77]. A recent study by Nozue *et al.* [77] suggests that PIF5 is a modulator of auxin signaling and that PIF4 and PIF5 regulate auxin sensitivity to control hypocotyl growth.

There are several possible mechanisms by which transcriptional auxin signaling may promote growth either by feeding into a PIF4/5-mediated pathway or acting independently. First, auxin might promote PIF4/5 activity by inducing *PIF4/5* transcription during the day; second, auxin might indirectly promote PIF4/5 activity by stimulating GA synthesis consequently degrading the DELLA repressors of the PIFs; third, auxin might act independently of PIF4/5 and regulate transcription of PIF4/5 targets during the day; last, auxin might act independently of the PIFs and regulate PIF4/5-independent growth genes. We addressed the first possibility by analyzing our microarray data. We did not detect a transcriptional response to auxin for the *PIF4/5* genes, suggesting that auxin either enhances residual PIF activity that may be present during the day, or acts in parallel to promote elongation independently of these proteins during the day.

**Figure 6:**
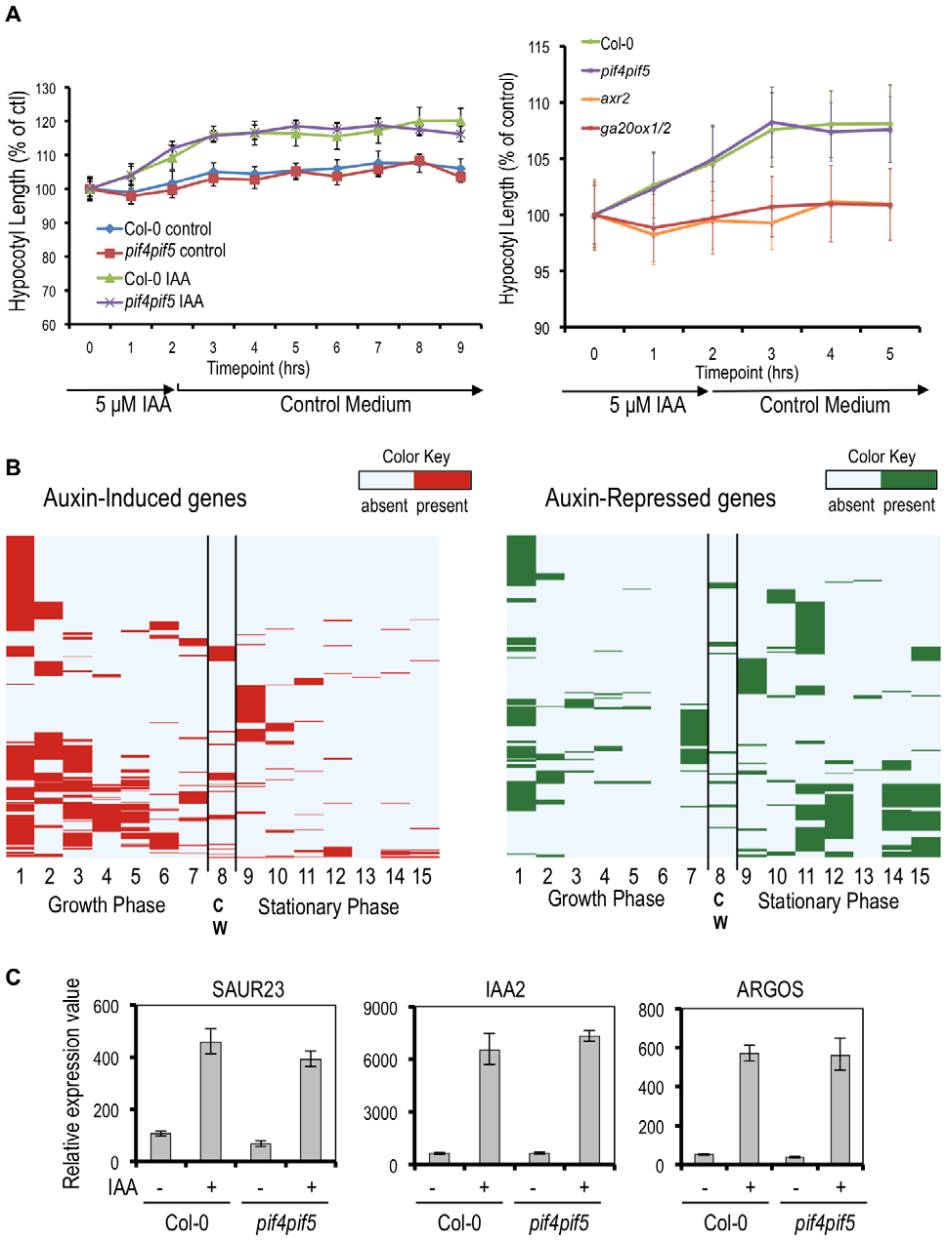
Auxin promotes hypocotyl elongation through PIF-dependent and –independent pathways. PIF4 and PIF5 are not required for transient auxin response. Average hypocotyl length of wild-type (Col-0) or *pif4pif5* mutant seedlings treated with 5 µM IAA for two hours was measured each hour for 7 hours. Error bars indicate standard error. Hypocotyl length at each time point is shown as a percentage of length at time 0. (B) Auxin-responsive genes are associated with growth conditions. Picloram-induced genes are also induced in the dark (column 1, up in WTD) and during growth (2, upG; 6, upG PIF4/5), and repressed by light (3, down in WTRc; 7, DL) and in the pifq mutant (4, down in pifqR1; 5, down in pifqD). Picloram-repressed genes are also repressed in the d*ark (colu*mn 9, down in WTD) and upregulated by light (10, upL; 15 up in WTRc), during stationary phase (11, upS; 13 Nozue upS PIF4/5), and in the pifq mutant (12, up in pifqR1; 14, up in pifqD) (see Methods, Table 1 and Table S6 for complete description of array conditions shown). CW indicates genes associated with cell wall metabolism (column 8). (C) PIF4/5-dependent genes are regulated by auxin in seedlings. Wild-type (Col-0) or *pif4pif5* mutant 5-day-old seedlin*gs were t*reated with 5 µM IAA or a solvent control for 2 hours and used for RNA isolation. Expression value of each gene shown, relative to a control gene, was determined by qRT-PCR.

We asked whether auxin, GA, and PIF4/5 are required for the initial growth response to a pulse of auxin using a time course elongation assay done during the day. A 2-hour auxin treatment led to an increase in hypocotyl length in wild-type seedlings within 2 hours (Fig. 6A). The response of *pif4pif5* mutant seedlings was indistinguishable from that of wild type, suggesting that this initial growth response does not require PIF4/5 protein. This result is not surprising given that PIF4 and PIF5 are rapidly degraded by a phyb-dependent mechanism and transcriptionally inhibited by the DELLAs during the day, and so are unlikely to be required for daytime growth [37]. In contrast, both the *ga20ox2* double mutant and the *axr2-1* are completely resistant to auxin in this assay (Fig. S4B). These results suggest that both auxin and GA are required for the initial growth response.

**Table 1:** Microarray data selected for comparison to auxin-regulated gene sets.

Reference	GEO ID	Conditions analyzed
Leivar et al. 2009	GSE17159	wild-type *vs. pifq* (*pif1 pif2 pif3 pif4 pif5* mutant) 2 day dark
		wild-type *vs. pifq* 2 day dark plus 1 hr red light
		wild-type 2 day dark treatment *vs. wild-type* 2 day red light
		wild-type seed *vs.* wild-type 2 day dark
Nozue et al. 2011	GSE21684	UpG PIF4/5 - Genes up in growth phase and PIF4/5 Dependent
		UpG - Genes up in stationary phase and PIF4/5 independent
		upS PIF4/5 - Genes up in stationary phase and PIF4/5 dependent
		upS - Genes up in stationary phase and PIF4/5 independent
Ma et al. 2005	GSE14648	6 day old light grown hypocotyls
		6 day old dark grown hypocotyls

To address whether the transcriptional targets of auxin signaling are also PIF targets, we performed an extensive comparison of our auxin-responsive cell expansion data set with existing growth-related microarray datasets. Nozue *et al.* [77] describe a series of global expression analyses in the *pif4pif5* mutant to classify sets of “growth” and “stationary” phase genes that are PIF4/5-dependent or -independent. Using the resulting gene lists as well as datasets obtained using various light conditions in wild type and a *pif1 pif3 pif4 pif5 PIF* quadruple mutant (*pifq*) [78], we compared our gene lists to the growth-regulated genes identified in these selected arrays. For a description of the arrays selected and the method of comparison see Methods, Table 1 and Table S6. We compared our auxin-induced and auxin-repressed gene lists to each array dataset and identified 490 auxin-induced genes and 270 auxin-repressed genes also presented in these growth datasets. We converted these results into a matrix in which each row represents an auxin-responsive gene from our list, and each column represents a microarray condition (Table S6). We then used hierarchical clustering to generate maps of each matrix. We divided each map into ‘growth’ and ‘stationary’ sections to reflect the conditions with which regulation of each gene is associated, as described by Nozue et al. [77] (Fig. 6B). We also included a column of genes associated with cell wall reorganization, ‘CW’ [79].

A pattern that emerges from our matrix maps is that many picloram-induced genes are co-regulated by conditions where growth is occurring. We found that 46% of our auxin-induced genes are induced in wild type 2-day-old seedlings grown in the dark when compared to light-grown seedlings (Fig. 6B column 1), and 21% are repressed by a 2-day red light treatment that inhibits hypocotyl elongation (Fig. 6B column 3). Similarly, the overlap between stationary phase genes and auxin-repressed genes is greater (the sum of values in columns 9–15 is 348 for 269 genes) than between stationary phase genes and auxin-induced genes (the sum of values in columns 9–15 is 191 for 490 genes) (Fig. 6B, left and right maps, columns 11,12,14,15). Therefore, our auxin-induced gene list consists at least in part of genes that are associated with growth, such as *ARGOS* (AT3G59900) and *ARGOS-LIKE* (AT2G44080)] [80], [81], *LONGIFOLIA1* (AT5G15580) and *LNG2* (AT3G02170) [82] and several *EXPANSIN* and *EXPANSIN-LIKE* genes (Table S6) [83], [84], [85].

The matrix maps highlight a significant overlap between PIF-regulated genes and auxin targets in elongating hypocotyls (Fig. 6B columns 3–6). This is consistent with previous results from Nozue et al. [77] that show that auxin-regulated genes are overrepresented among genes differentially expressed between *pif4pif5* double mutant and wild type plants. Not surprisingly, genes in this category include genes associated with GA pathways including gibberellin biosynthesis genes *GA3OX1* and *GA2OX8*, the GA repressor *RGL1*, *PIF3-LIKE2* (AT3G62090) and SOMNUS (AT1G03790), a germination gene downstream of *PIL5* (AT2G20180). Of the 81 genes defined by Nozue et al. [77] as upregulated by growth and PIF4- or PIF5-dependent, 38 are also classified in that study as auxin regulated. Of these 38, 35 are in our auxin-induced list. Our auxin-induced list also includes an additional 17 PIF4/5-dependent genes not classified by Nozue *et al.* as auxin-responsive [77].

These findings raise the question of whether auxin regulates PIF target genes through induction of GA biosynthesis and consequent PIF activation, through a GA-independent PIF process, or through a PIF-independent mechanism. We predicted that a set of PIF4/5-dependent growth-associated genes might be auxin-regulated in the absence of PIF activity, since the hypocotyl growth response to the transient auxin treatment during the day did not require PIF4/5 (Fig. 6A). We tested the response of a subset of growth-associated genes, including *SAUR23* (AT5G18060), *IAA2* (AT3G23030) and *ARGOS*, to auxin using qRT-PCR. We found that each of these three genes was induced by a 2 hr IAA treatment in *pif4pif5* double mutant seedlings (Fig. 6C). This suggests that these genes are directly regulated by auxin. This has been confirmed for *IAA2*, which is rapidly induced by auxin in the presence of cyclohexamide [86]. The fact that these three genes are PIF-dependent in growth promoting-conditions can be explained by the recent discovery that PIF4/5 directly regulates auxin biosynthesis at elevated temperature [39], [42], [77], [87], [88]. Genes in this category may be direct auxin targets whose expression in growth-promoting conditions, such as elevated temperature, is dependent upon PIF regulation of auxin biosynthesis. However, we do not rule out the possibility that such genes may also be directly regulated by the PIF family in some conditions. Thus, our results support a growth model in which a number of important cell expansion-associated genes are common targets of multiple growth-promoting pathways.

Finally, our analysis also revealed overlap between auxin-responsive genes and growth-upregulated genes that are PIF4/5-independent. More than 200 of our auxin-induced genes are in this category. While this group predictably includes auxin transport (*e.g. PINOID-BINDING PROTEIN1*, AT5G54490; *TOUCH3*, AT2G41100 [89] and signaling factors (*IAA7*, AT3G23050; *IAA5*, AT1G15580), genes in the GA pathway (*GAI*, AT1G14920; *GA20OX2*, AT5G51810), ethylene pathway (*ETHYLENE RESPONSE 2*, AT3G23150; *ETHYLENE RESPONSE SENSOR 1*, AT2G40940; *ERS2*, AT1G04310), and brassinosteroid pathway (*BES1/BZR1 HOMOLOG 2*, AT4G36780; *BRASSINAZOLE-RESISTANT 1*, AT1G75080) are also present. Additionally, several genes with roles in cell wall metabolism are present, including *XYLOGLUCAN ENDOTRANSGLUCOSYLASE/HYDROLASE 16* (AT3G23730), *XTH17* (AT1G65310) and *XTH8* (AT1G11545), *CELLULOSE SYNTHASE-LIKE D3* (AT3G03050), and *CELLULOSE SYNTHASE-INTERACTIVE PROTEIN 1* (AT2G22125). Together, these genes are candidate direct auxin targets involved in growth and in cross-talk with other signaling pathways.

### Auxin Regulates Additional Candidate Cell Expansion Genes

In our auxin-responsive gene list, 81 genes we identified as auxin-induced and 70 genes we identified as auxin-repressed are interrogated by the NimbleChip but not by the Affymetrix ATH1 chip (ftp://ftp.arabidopsis.org/home/tair/Microarrays/Affymetrix/). These genes are presented in Table S4. Several genes among these have predicted functions in cell expansion, including *BREVIS RADIX* (AT1G31880), which promotes leaf, root and shoot growth [90], *KIDARI* (AT1G26945), which promotes shoot elongation downstream of GA [91], and *PAR2* (AT3G58850), a transcription factor induced during the shade avoidance response [92]. Due to a lack of available microarray data, we did not further explore the expression profiles or functions of these genes. However, we confirmed auxin-responsiveness of *PAR2* as well as of *CTR1* (AT5G03730) and *BRIL* (AT1G55610) (new candidate growth genes that are not represented on the ATH1 chip) in seedlings using qRT-PCR (Fig. S5).

### Concluding Remarks

**Figure 7:**
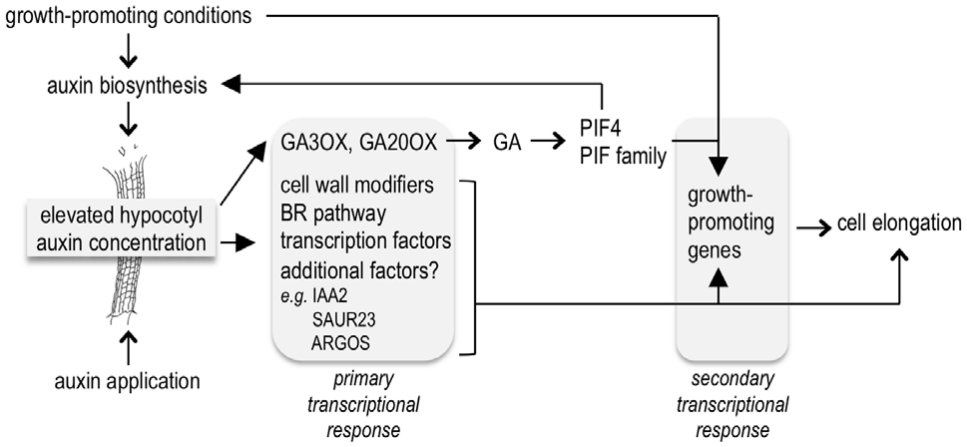
Model for the transcriptional auxin response preceding hypocotyl elongation. Auxin levels in hypocotyl tissue elevate in response to growth-promoting conditions or exogenous auxin, which activates transcriptional auxin signaling. Early auxin-responsive genes include those encoding GA oxidases, cell wall modifying enzymes, and other factors that may contribute directly to cell elongation or regulate expression of additional growth-promoting genes. These pathways may *be reinf*orced by activity of PIF4 and PIF5, which are liberated from DELLA repression due to auxin-mediated modulation of GA levels. In growth-promoting conditions, auxin-responsive genes may be PIF-dependent due to PIF regulation of auxin biosynthesis [39], [42], [77], [87].

Our hypocotyl sampling approach enabled us to detect auxin-responsive growth-associated genes that have not been detected in many whole seedling arrays. It is possible that the large number of genes in our auxin-responsive lists that were not found in the MASTA analysis or the comparison with the Nemhauser *et al.* [61] and Stepanova *et al.* [62] datasets represent genes that are auxin-responsive in a specific spatio-temporal pattern that is masked in experimental designs using diverse tissue homogenates. Results from this study emphasize the value of tissue-specific analyses when addressing a particular developmental question. We have uncovered a large set of auxin-regulated genes that are expressed in elongating hypocotyls, including several GA biosynthesis enzymes (Fig. 7). Our results suggest that auxin regulates GA biosynthesis to release DELLA-dependent growth repression [37]. Genetic analyses confirmed the importance of auxin-GA cross-talk for a complete hypocotyl growth response, a process that has also been reported in pea [93]. However, we also demonstrated that regulation of GA is not the only mechanism for auxin-stimulated hypocotyl growth and an independent pathway is required for optimal response. Interestingly, auxin-GA interplay is also involved in tropic hypocotyl growth, although in these processes GA is required to attenuate growth through repression of auxin signaling [94]. It will be important for a complete understanding of hormone-regulated growth to assign downstream growth genes to specific hormone pathways or identify mechanisms and conditions in which these genes are downstream of multiple signaling pathways, as we have proposed for *IAA2*, *ARGOS*, and other genes.

Under normal growth conditions, the circadian clock maintains diurnal hypocotyl growth by gating auxin response primarily through PIF4 and PIF5 [39], [41], [42]. However, various stress conditions cause plants to stimulate rapid changes in growth during the day in order to survive. For example, rapid flooding causes changes in hormone levels within 1 h of submergence. Studies in *Rumex palustris* have revealed the importance of ethylene, IAA and GA in stimulating stem elongation following submergence due to rapid flooding [95]. Our hypocotyl transcriptome analysis was performed under conditions that mimic a rapid increase in auxin levels during the day leading to a hypocotyl elongation response. We have identified many cell elongation genes that are known to be growth-associated but have not been previously described as auxin-responsive (Fig. 7). A subset of these genes is described as being PIF4/5-dependent and we would expect this regulation to be active during normal hypocotyl growth conditions during the night when the PIFs are present [39], [42]. However, our results suggest that auxin activates these genes in the absence of PIF4/5 suggesting that auxin promotes hypocotyl growth by an independent pathway during the day. It will be interesting to determine how the activities and expression patterns of these genes are important for the extent and timing of hypocotyl growth. Among our auxin-responsive gene list are genes involved in cell wall biogenesis and secretory pathways known to be important for cell elongation. Using the hypocotyl tissue-specific approach and the NimbleChip, we have also uncovered additional hypocotyl growth genes that may also be important for other cell expansion dependent processes such as petiole growth. With this study we present a transcriptional framework for rapid stimulation of hypocotyl elongation during the day, independent of PIF4 and PIF5, and we provide a genomic basis for the model that auxin, GA, and the PIFs have overlapping roles in regulating growth.

## Materials and Methods

### Plant Material

*Arabidopsis thaliana* mutants and transgenic lines used in this study were all in the *Columbia* (Col-0) ecotype. Mutants *msg2-1* [48], , *slr-1* [49], and *pif4-101/pif5-1* [36] were described previously. *tir1-1, afb1-3, afb2-3, afb3-4, afb4-2, afb5-5* and higher-order combinations among these mutants were described previously [27], [97]. *RGA::GFP-RGA* (CS16360) was obtained from the Arabidopsis Biological Resource Center at The Ohio State University, and the *ga20ox1ox2* mutant [98] was a generous gift from Peter Hedden. For hormone treatment assays and RNA isolations, seeds were plated on ½× Murashige-Skoog medium containing 1% sucrose and 1% agar, and stratified 2–4 days in the dark at 4°C.

### Hypocotyl Growth Assays and Imaging

Seedlings were grown under long day photoperiods (16 h light/8 h dark) at 23°C unless otherwise indicated, with white light intensity of ∼80 µmol/m^2^/s. For treatment assays and RNA isolations, 5-day-old seedlings were transferred to plates containing the chemical being tested or the solvent control (DMSO was used for picloram; ethanol was used for IAA, GA_3_, paclobutrazol and NPA) for an additional 48 hours unless otherwise stated. Hypocotyl images were taken using a Nikon SMZ1500 dissecting scope and all measurements were done using ImageJ software. Data shown represent an average of at least 10 seedlings per treatment; error bars represent standard error.

Seedlings used for visualization of RGA-GFP were grown in long day conditions, and RGA-GFP fluorescence at time zero was analyzed four hours after subjective dawn in two day-old seedlings. Seedlings were then submerged in liquid medium (½× Murashige-Skoog medium containing 1% sucrose) containing the chemical being tested or the solvent control (ethanol) for an additional 2–24 hours, and a subset of seedlings was removed from treatment and imaged at the time points indicated. GFP fluorescence in pRGA:RGA-GFP was visualized using a Nikon SMZ1500 dissecting scope.

### Transcriptome Experiments

#### Microarray “a”

Stratified seeds were plated on medium overlaid with sterilized nylon mesh (110 micron pore size; www.smallparts.com). Two hours after chamber lights came on, mesh rafts containing 5-day-old seedlings were transferred to medium containing 5 µM picloram or an equivalent volume of DMSO for 30 min or two hours. Hypocotyls were dissected over a 30-minute period and frozen in liquid nitrogen. Tissue samples were collected over several days and pooled into biological replicates containing at least 400 hypocotyls. RNA extractions were done using Trizol reagent (Sigma) followed by additional phenol extraction and ethanol precipitation steps. mRNA was amplified using the MessageAmp II aRNA Amplification kit (Ambion) and the manufacturer’s protocol. Labeled cDNA was prepared from aRNA using the Superscript ds cDNA synthesis kit (Invitrogen), Cy3- and Cy5-labeled random nonomers (TriLink) and Klenow fragment (Promega). Samples representing three biological replicates were selected for hybridization to the 4-plex NimbleGen chip at the Center for Genomics and Bioinformatics at Indiana University. Experiment ‘a’ was hybridized to the NimbleGen 4-plex chip using dual-color labeling.

#### Microarray “b”

Seedlings were grown and treated as described for experiment ‘a’; however, roughly 700 hypocotyls were included in each biological replicate to avoid RNA amplification. RNA extractions were performed using the Invitrogen PureLink RNA mini Kit. Three biological replicates were sampled and used for cDNA synthesis and hybridization to the 12-plex NimbleGen chip according to manufacturer’s instructions. Experiment ‘b’ was hybridized to the NimbleGen 12-plex chip using single-color labeling. Microarray ‘b’ was carried out at the GeneChip™ Microarray Core facility at the University of California San Diego.

### Transcriptome Analysis

All microarray analysis was done using R (R Development Core Team (2011), http://www.R-project.org/) and Bioconductor [99]. Microarray ‘a’ was annotated based on the TAIR8 version and ‘b’ was annotated based on TAIR10. Annotation packages were built with pdInfoBuilder using raw data files (.xys) along with a Nimblegen microarray design file (.ndf). All microarrays were RMA normalized using *oligo* in R with this annotation package. Normalized data for array ‘a’ and ‘b’ were analyzed independently using a linear model method [56] performed in the LIMMA package in R. Differentially expressed genes were chosen based on an Empirical Bayes method and an FDR of less than 0.05. To identify a statistically significant list of differentially expressed genes from microarray ‘a’ and ‘b’, Rank Product method was used due to the difficulty comparing datasets derived from independent experiments [58]. As shown in Vert et al. [58], this method [60], [99] often outperforms the linear model when comparing microarray experiments derived from different laboratories. There are several advantages to this method, including the use of pre-processed data, eliminating the requirement for normalizing heterogeneous data together that will often retain ‘lab-effects’ [58]. The Rank Product method includes fewer assumptions under the model, accounts for multiple sources of datasets and performs better with noisy data or a low number of replicates. The expression values are transformed into ranks allowing for the integration of datasets from a variety of platforms [59], [60]. Genes or splice forms that were not present on both chips were removed from the analysis. Upregulated and downregulated gene lists from RankProd were used for the comparisons described below. Microarray data have been deposited in NCBI’s Gene Expression Omnibus [100] and are accessible through GEO Series accession number GSE37217 (www.ncbi.nlm.nih.gov/geo/query/acc.cgi?acc=GSE37217).

### Array Comparisons

The MASTA package available from the BAR website (www.bar.utoronto.ca) was used to compare RankProd-generated lists with the 7 IAA wild-type treatment arrays included in the MASTA package. The IAA root treatment data from Stepanova *et al.* [62] was downloaded from the Gene Expression Omnibus (GEO) database (www.ncbi.nlm.nih.gov/geo/). CEL files were RMA normalized using the Affymetrix package and input into RankProd. Differentially expressed genes with an FDR less than 0.05 were selected and compared to the picloram-responsive gene lists. For the Nemhauser *et al.* [61] comparisons the genes defined by the authors as auxin-responsive were used.

Microarrays selected for the growth gene comparisons are listed in Table 1, Tables S6 and S7. CEL files were not available for all of the arrays selected; picloram-responsive genes identified in this study were compared with genes defined as differentially expressed according to the publication associated with the data in GEO. Matrices were generated with the picloram-induced and –repressed genes in which each row represents an auxin-responsive gene and each column represents a treatment condition from the array being compared. Genes were assigned a value of 1 if defined as differentially expressed in the associated publication, or a value of 0 if absent from the data set. The resulting matrix was used to generate a hierarchical clustered based map in R. Columns were manually arranged based on conditions where growth is occurring (growth phase) or inhibited (stationary phase). The middle column in each map (cw) includes genes that were defined by Jamet *et al.* [79] as being involved in cell wall biogenesis or secretory pathways likely important for cell wall expansion.

### Quantitative RT-PCR

RNA samples collected from hypocotyl and whole seedling tissue were obtained from tissue frozen in liquid N2 using the INVITROGEN PureLink RNA minikit. RNA yield and quality was quantified using the Thermo Scientific NanoDrop 2000. Equal amounts of RNA were pre-treated with DNase using the DNA-free Kit (Ambion) according to manufacturer’s instructions and used to generate cDNA with SuperScript III First-Strand Synthesis (Invitrogen) with 20-mer oligo(dT) primers. Quantitative RT-PCR was done with SyBR green and the primers listed in Table S8. Primer pairs were evaluated for specificity and efficiency using three serial dilutions of cDNA using the CFX96™ Real-Time PCR Detection System (Biorad). Data were normalized to the reference gene PP2AA3 [101] according to the ΔΔCt method [102]. Primers were designed using QuantPrime [103]. Experiments with hypocotyl or seedling tissue were done with two biological replicates and three technical replicates.

## Supporting Information

Figure S1 **Picloram and IAA share transcriptional targets.** (**A**) Microarray analysis of picloram-regulated genes in hypocotyls and IAA-regulated genes in seedlings or roots identified common target genes. Venn diagrams of auxin-upregulated genes in IAA-treated materials (IAA-up) and hypocotyls of picloram-treated seedlings (Pic-up) are shown. Numbers of genes identified in common are shown in the overlap sections of each diagram. (B) Auxin marker genes are picloram-responsive in hypocotyls from wild-type, but not *afb5-5* mutant, seedlings. Hierarchical clustering result of IAA marker gene expression in hypocotyls of picloram-treated or control wild-type (Col-0) or *afb5-5* (*afb5)* seedlings, as determined by analysis of microarray data using ArrayStar, is shown. (C) IAA marker genes are regulated by picloram in hypocotyls of picloram-treated seedlings. Wild-type (Col-0) or *afb5-5* mutant seedlings were treated with picloram or a solvent control for 2 hours and used for hypocotyl dissection and RNA isolation. Expression value of each gene shown, relative to a control gene, was determined by qRT-PCR.
(TIF)

Figure S2 **Auxin response elements are overrepresented in picloram-responsive promoters.** Statistical significance of overrepresentation of each AuxRE-containing sequence element (p-value) is plotted on the x-axis; the number of promoters containing the element is plotted on the y-axis. Overrepresented sequences were identified using ELEMENT [105].
(TIF)

Figure S3 **GO terms newly associated with auxin-responsive transcription.** Overrepresented GO terms and enrichment scores were identified using GOMiner [106]. Only GO terms not overrepresented in the AtGenExpress datasets [61] are shown.
(TIF)

Figure S4 **GA and auxin act independently and interdependently to regulate hypocotyl elongation.** (**A**) Auxin signaling mutants are partially restored by treatment with GA_3_. Average hypocotyl length of wild-type seedlings or the indicated mutants grown in long days and treated with 50 µM GA_3_ was determined following 48 hours of treatment. Statistical significance was determined using a Tukey HSD post hoc comparison among the means on the analysis of variance using type III sums of squares (p<0.05). Error bars indicate standard error. (b)The *axr2-1* and *ga20ox2* double mutant are deficient in transient auxin response. Average hypocotyl length of wild-type and mutant seedlings treated with 5 µM IAA for two hours was measured each hour for 7 hours. Hypocotyl length at each time point is shown as a percentage of length at time 0. Error bars indicate standard error.
(TIF)

Figure S5 PIF4/5-independent genes are regulated by auxin in seedlings. Wild-type seedlings were treated with IAA or a solvent control for 2 hours and used for RNA isolation. Expression value of each gene shown, relative to a control gene, was determined by qRT-PCR.
(TIF)

Table S1 Microarray experimental design.
(XLSX)

Table S2 Genes auxin-responsive at 30 minutes.
(XLSX)

Table S3 Genes auxin-responsive at 120 minutes.
(XLSX)

Table S4 Newly identified auxin-responsive genes.
(XLSX)

Table S5 GO terms associated with auxin-responsive gene lists.
(XLSX)

Table S6 Genes and microarray datasets presented in Figure 7B, auxin-induced genes.
(XLSX)

Table S7 Genes and microarray datasets presented in Figure 7B, auxin-repressed genes.
(XLSX)

Table S8 Primer sequences used for quantitative RT-PCR.
(XLSX)
