# Supplementary figures and images for: Hypocotyl Transcriptome Reveals Auxin Regulation of Growth-Promoting Genes through GA-Dependent and -Independent Pathways
Source: PLoS One. 2012 May 9;7(5):e36210. doi: 10.1371/journal.pone.0036210 (PMC3348943; doi:10.1371/journal.pone.0036210)

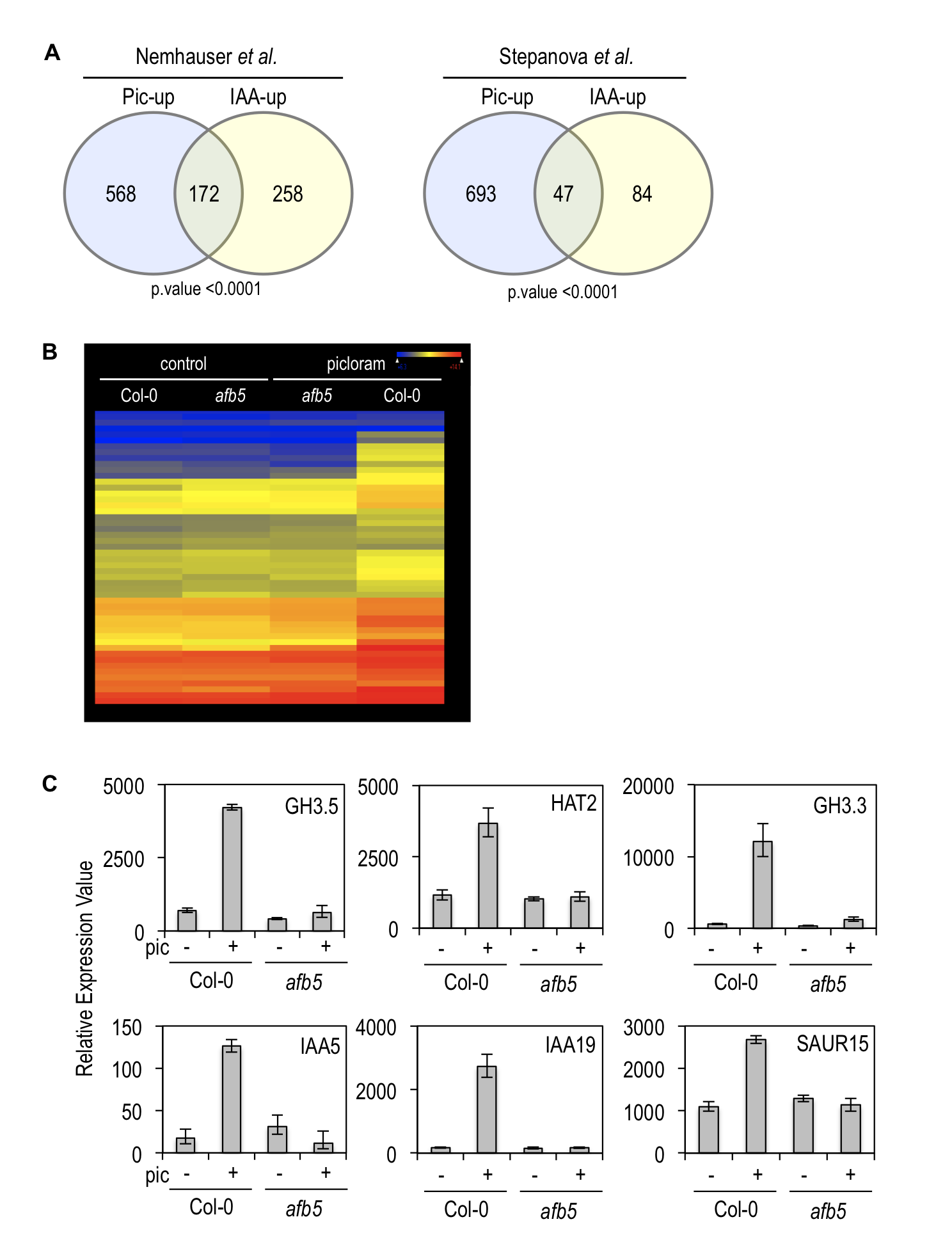

Supplement: Figure S1 — Picloram and IAA share transcriptional targets. (A) Microarray analysis of picloram-regulated genes in hypocotyls and IAA-regulated genes in seedlings or roots identified common target genes. Venn diagrams of auxin-upregulated genes in IAA-treated materials (IAA-up) and hypocotyls of picloram-treated seedlings (Pic-up) are shown. Numbers of genes identified in common are shown in the overlap sections of each diagram. (B) Auxin marker genes are picloram-responsive in hypocotyls from wild-type, but not afb5-5 mutant, seedlings. Hierarchical clustering result of IAA marker gene expression in hypocotyls of picloram-treated or control wild-type (Col-0) or afb5-5 (afb5) seedlings, as determined by analysis of microarray data using ArrayStar, is shown. (C) IAA marker genes are regulated by picloram in hypocotyls of picloram-treated seedlings. Wild-type (Col-0) or afb5-5 mutant seedlings were treated with picloram or a solvent control for 2 hours and used for hypocotyl dissection and RNA isolation. Expression value of each gene shown, relative to a control gene, was determined by qRT-PCR. (TIF) [file pone.0036210.s001.tif]

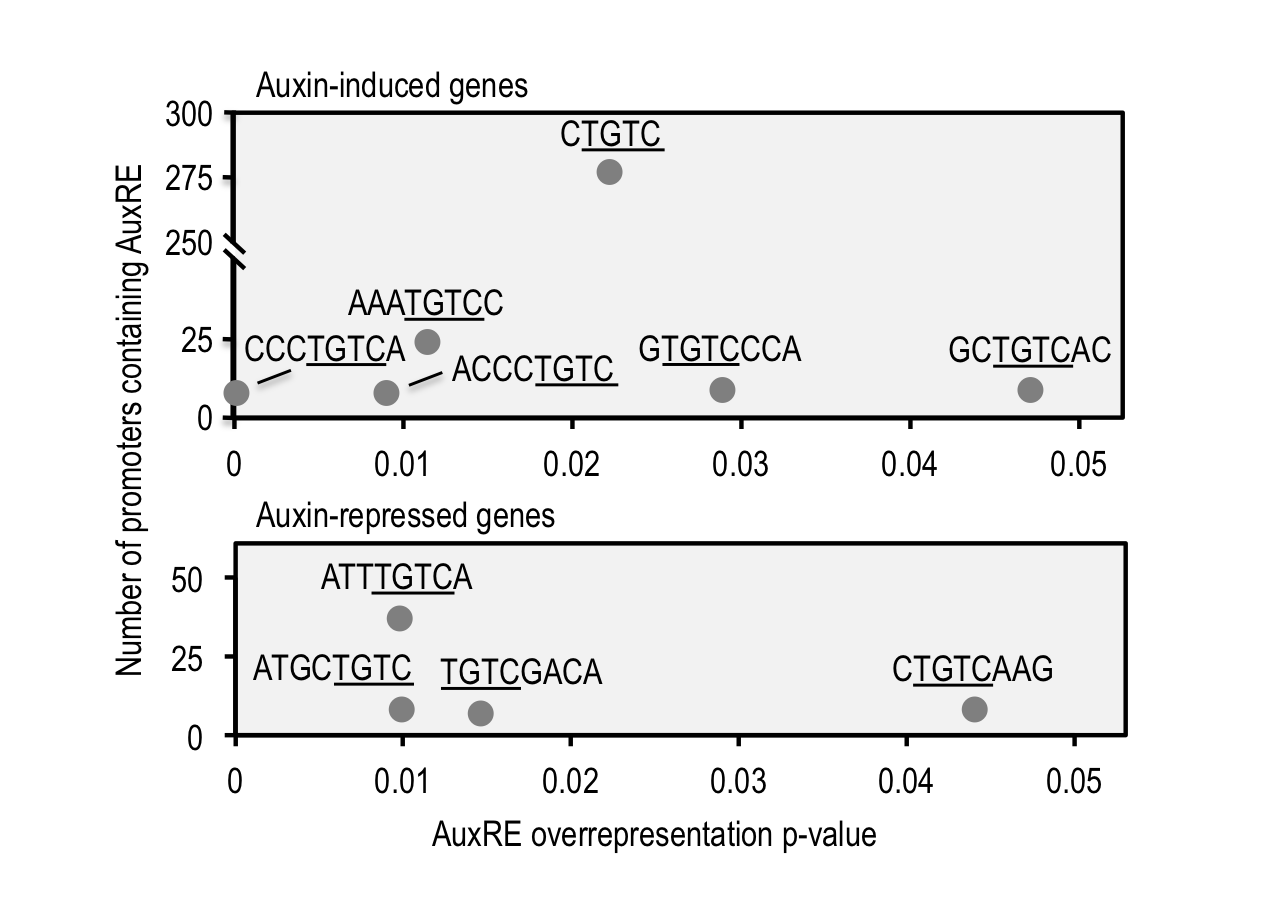

Supplement: Figure S2 — Auxin response elements are overrepresented in picloram-responsive promoters. Statistical significance of overrepresentation of each AuxRE-containing sequence element (p-value) is plotted on the x-axis; the number of promoters containing the element is plotted on the y-axis. Overrepresented sequences were identified using ELEMENT [105]. (TIF) [file pone.0036210.s002.tif]

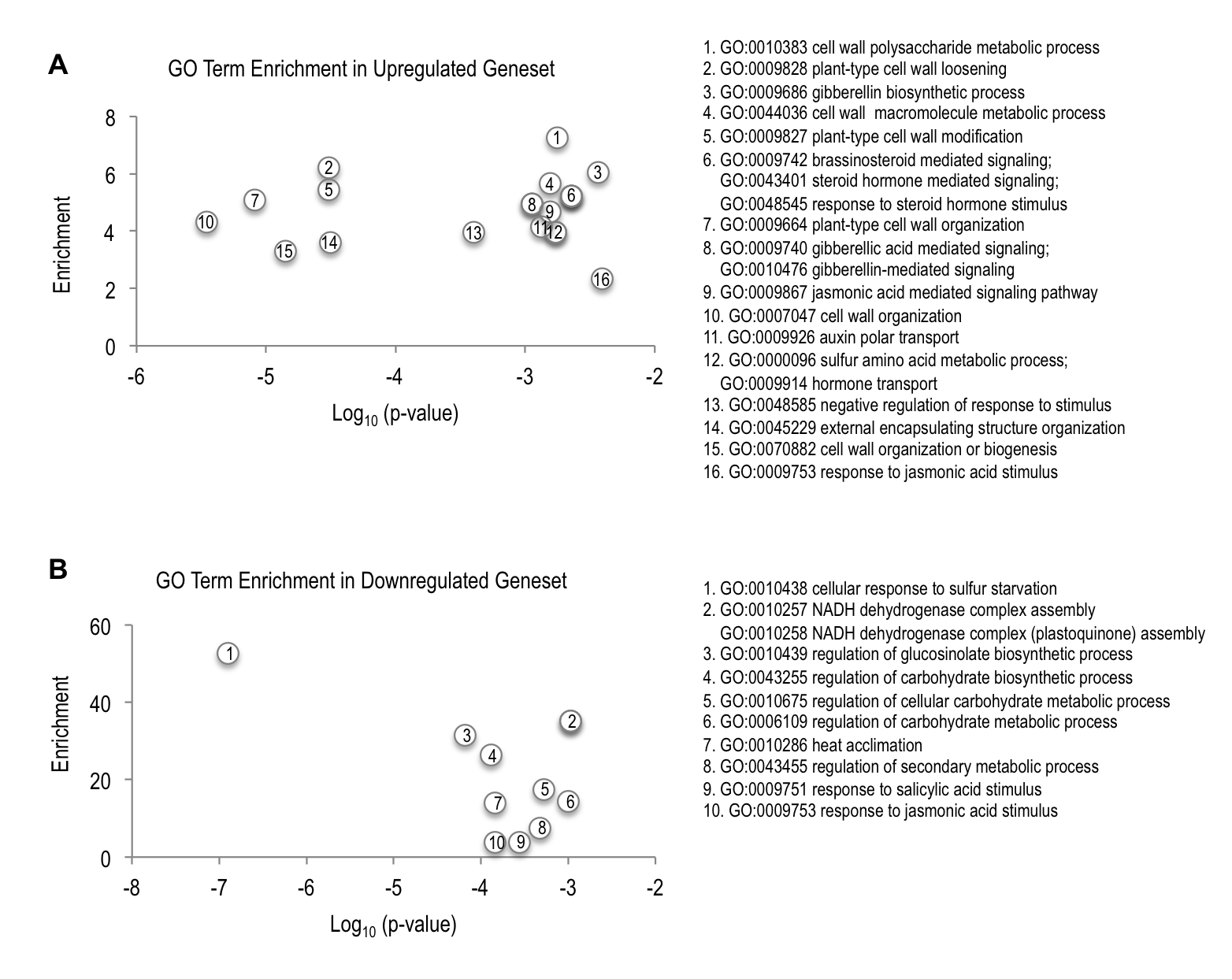

Supplement: Figure S3 — GO terms newly associated with auxin-responsive transcription. Overrepresented GO terms and enrichment scores were identified using GOMiner [106]. Only GO terms not overrepresented in the AtGenExpress datasets [61] are shown. (TIF) [file pone.0036210.s003.tif]

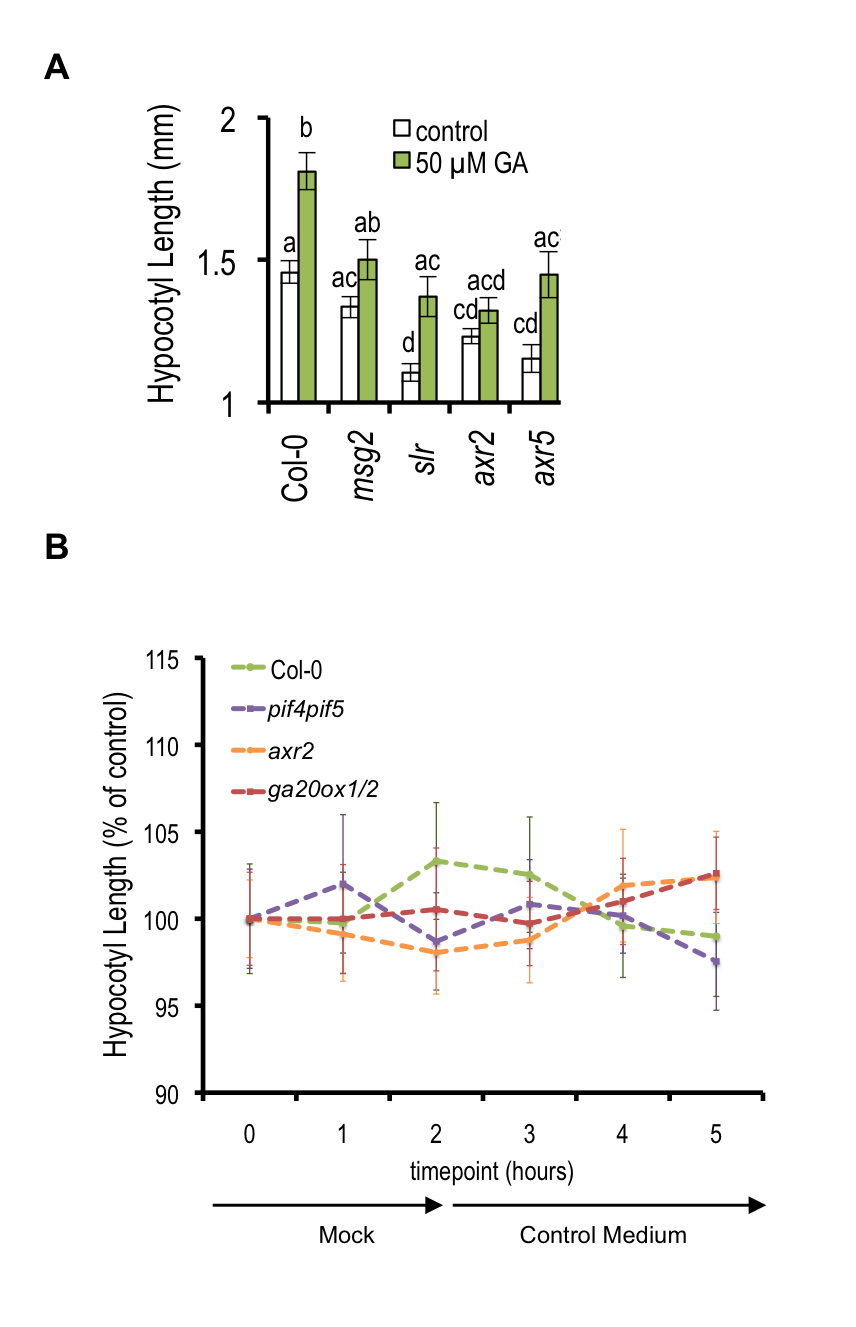

Supplement: Figure S4 — GA and auxin act independently and interdependently to regulate hypocotyl elongation. (A) Auxin signaling mutants are partially restored by treatment with GA3. Average hypocotyl length of wild-type seedlings or the indicated mutants grown in long days and treated with 50 µM GA3 was determined following 48 hours of treatment. Statistical significance was determined using a Tukey HSD post hoc comparison among the means on the analysis of variance using type III sums of squares (p<0.05). Error bars indicate standard error. (b)The axr2-1 and ga20ox2 double mutant are deficient in transient auxin response. Average hypocotyl length of wild-type and mutant seedlings treated with 5 µM IAA for two hours was measured each hour for 7 hours. Hypocotyl length at each time point is shown as a percentage of length at time 0. Error bars indicate standard error. (TIF) [file pone.0036210.s004.tif]

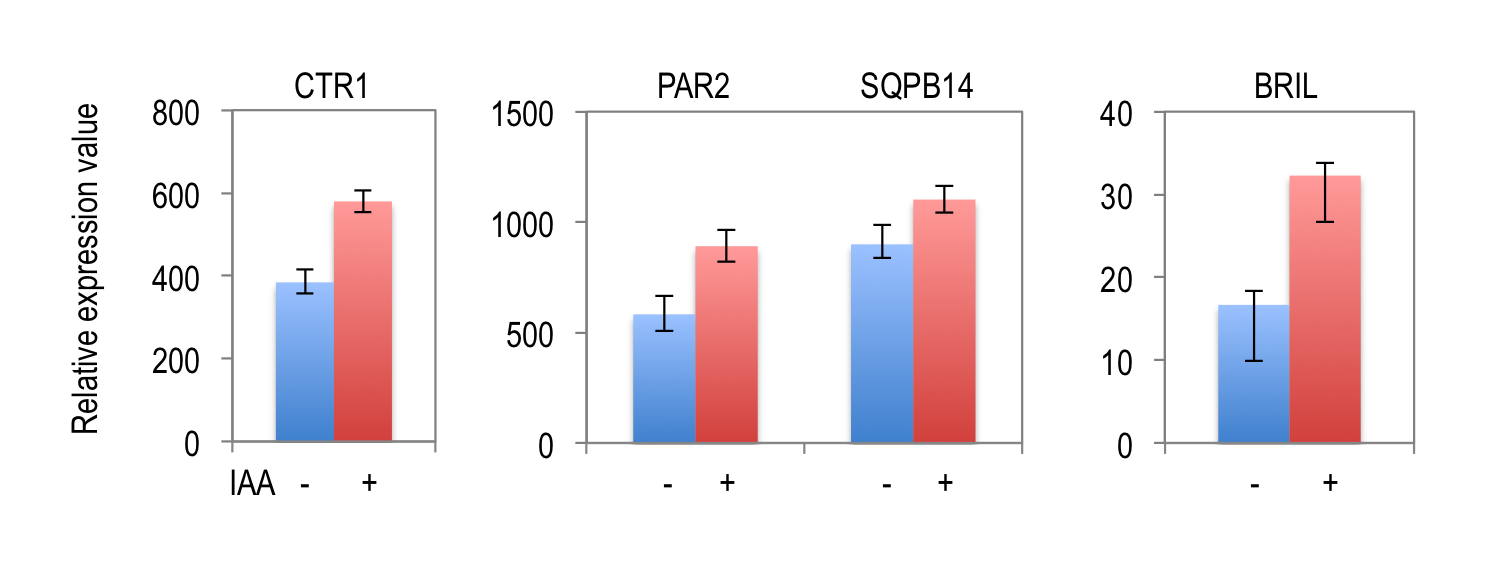

Supplement: Figure S5 — PIF4/5-independent genes are regulated by auxin in seedlings. Wild-type seedlings were treated with IAA or a solvent control for 2 hours and used for RNA isolation. Expression value of each gene shown, relative to a control gene, was determined by qRT-PCR. (TIF) [file pone.0036210.s005.tif]
